# A self-inactivating retrovector incorporating the IL-2 promoter for activation-induced transgene expression in genetically engineered T-cells

**DOI:** 10.1186/1743-422X-3-97

**Published:** 2006-11-21

**Authors:** Diana E Jaalouk, Laurence Lejeune, Clément Couture, Jacques Galipeau

**Affiliations:** 1 Lady Davis Institute for Medical Research, McGill University, Montreal, Quebec, Canada; 2 Division of Hematology/Oncology, Jewish General Hospital, McGill University, Montreal, Quebec, Canada; 3 The University of Texas M. D. Anderson Cancer Center, Unit 1374, P.O. Box 301439, Houston, Texas, USA

## Abstract

**Background:**

T-cell activation leads to signaling pathways that ultimately result in induction of gene transcription from the interleukin-2 (IL-2) promoter. We hypothesized that the IL-2 promoter or its synthetic derivatives can lead to T-cell specific, activation-induced transgene expression. Our objective was to develop a retroviral vector for stable and activation-induced transgene expression in T-lymphocytes.

**Results:**

First, we compared the transcriptional potency of the full-length IL-2 promoter with that of a synthetic promoter composed of 3 repeats of the Nuclear Factor of Activated T-Cells (NFAT) element following activation of transfected Jurkat T-cells expressing the large SV40 T antigen (Jurkat TAg). Although the NFAT3 promoter resulted in a stronger induction of luciferase reporter expression post stimulation, the basal levels of the IL-2 promoter-driven reporter expression were much lower indicating that the IL-2 promoter can serve as a more stringent activation-dependent promoter in T-cells. Based on this data, we generated a self-inactivating retroviral vector with the full-length human IL-2 promoter, namely SINIL-2pr that incorporated the enhanced green fluorescent protein (EGFP) fused to herpes simplex virus thymidine kinase as a reporter/suicide "bifunctional" gene. Subsequently, Vesicular Stomatitis Virus-G Protein pseudotyped retroparticles were generated for SINIL-2pr and used to transduce the Jurkat T-cell line and the ZAP-70-deficient P116 cell line. Flow cytometry analysis showed that EGFP expression was markedly enhanced post co-stimulation of the gene-modified cells with 1 μM ionomycin and 10 ng/ml phorbol 12-myristate 13-acetate (PMA). This activation-induced expression was abrogated when the cells were pretreated with 300 nM cyclosporin A.

**Conclusion:**

These results demonstrate that the SINIL-2pr retrovector leads to activation-inducible transgene expression in Jurkat T-cell lines. We propose that this design can be potentially exploited in several cellular immunotherapy applications.

## Background

T-cells have several features that render them attractive target cells for cell and gene therapy applications. In addition to their diverse and critical role in the immune system, T-cells are readily available from human peripheral blood, they can be easily isolated, activated, expanded to large numbers *in vitro*, and reinfused with a potential to circulate for a long time *in vivo*. Indeed, T-cells have been successfully utilized for adoptive immunotherapy of cancer, viral infections, and autoimmune diseases.

Since adoptive transfer of allogeneic T-cells has been associated with acute graft-versus-host disease (GVHD), a life-threatening complication initiated by the donor alloreactive T-cells against the normal host tissues, alternative approaches have been attempted to reduce the risk of GVHD while maintaining the beneficial aspects of this treatment modality. One scenario was to use allogeneic T-cells genetically-engineered to express the herpes simplex virus thymidine kinase (HSVTK) suicide gene that would lead to the depletion of the gene-modified T-cells following treatment with the drug ganciclovir if GVHD arises [1]. This strategy gave promising results in a number of clinical studies [2,3]. In a second approach, *in vitro *enriched antigen-specific T-cells were used instead of total allogeneic T-cell populations to mount antiviral or antitumour responses. Donor-derived cytomegalovirus (CMV)-specific cytotoxic T lymphocyte (CTL) clones were successfully used to treat patients suffering from CMV viral infection following marrow transplants [4]. Similarly, infusion of Epstein-Barr virus (EBV)-specific CTLs was used to prevent and to treat EBV lymphoma in immuno-compromised patients post BMT [5]. Interestingly, more recent approaches in cancer immunotherapy investigated the use of T-cells primed *in vitro *against minor histocompatibility antigens [6], or against tumor associated antigens [7,8].

Moreover, autologous T-cells genetically engineered to express antiviral transgenes have been used for the treatment of HIV [9,10]. Similar studies investigated the use of autologous T-cells engineered to express anti-inflammatory cytokines for the treatment of autoimmune diseases [11,12]. The success of these approaches is dependent on the stable transfer of the therapeutic gene into all infused T-cells and its subsequent expression upon T-cell activation. On the other hand, successful use of antigen-specific T-cells is dependent upon their rapid *in vitro *selection and enrichment. Therefore, stable and selective expression of a gene marker in activated antigen-specific T-cells would improve their therapeutic utility.

Integrating retroviral vectors have been successfully used for stable expression of transgenes in T-cells [2,10,13]. Recently, the development of new retroviral pseudotypes and improved transduction protocols have made it possible to gene modify T-cells with high efficiency [14]. Furthermore, it was documented that the level of transgene expression can vary with the activation status of T-cells [15]. In fact, it has long been known that the enhancer machinery within the retroviral long terminal repeat (LTR) such as that of Moloney murine leukemia virus (MoMLV) incorporates elements that confer transcriptional preference to activated T-cells [16,17]. However, LTR-driven transgene expression is neither exclusive to the activated subset of gene-modified T-cells nor does it guarantee that all activated transduced T-cells express the transgene if the vector integrates in a transcriptionaly silent chromosomal background [18-20].

Activation of T-cells, physiologically by triggering of the T-cell receptor (TCR) complex or pharmacologically by using reagents such as ionomycin and PMA results in the production of IL-2 which plays a key role in T-cell proliferation and differentiation. Therefore, IL-2 gene transcription by induction of the IL-2 promoter is specific to activated T-cells [21]. Plasmid vectors in which reporter gene expression is under the control of the full-length IL-2 promoter or synthetic promoters composed of either single or multiple binding motifs within the IL-2 promoter have been used in many investigative studies of T-cell activation [22-24]. In these studies, reporter gene expression was induced following activation of the transfected T-cell lines. Hence, we propose that incorporation of the IL-2 promoter or its synthetic derivatives into a transcriptionaly silent retroviral vector would lead to stable and activation-induced transgene expression in T-cells. Our objective is to develop a retroviral vector for stable activation-induced transgene expression in T-lymphocytes.

In this study, we first compared the transcriptional potency and stringency of the full-length IL-2 promoter with that of a synthetic promoter composed of 3 NFAT elements following stimulation of T-cells. Transient transfections of Jurkat Tag cells with plasmids harboring either one of the two promoters upstream of a luciferase reporter showed that although the NFAT3 synthetic promoter resulted in a stronger induction of reporter expression following T-cell activation, the IL-2 promoter had a more stringent activation-dependent expression. Consequently, we tested if a self-inactivating retroviral vector incorporating the full-length IL-2 promoter namely SINIL-2 pr would serve as an integrated platform for stable and specific activation-induced transgene expression in T-cell lines.

## Results

### Activation-induced luciferase reporter expression in Jurkat TAg cells

**Figure 1:**
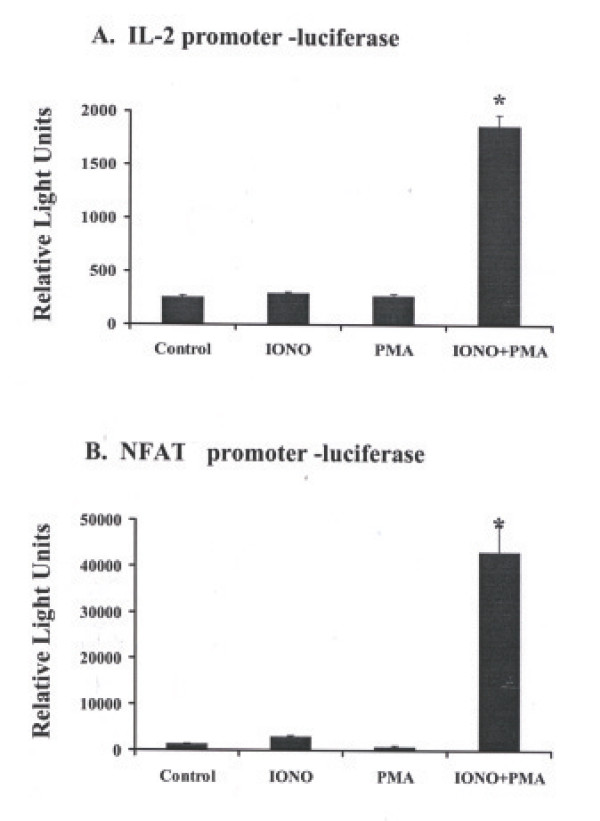
**Activation-induced luciferase reporter expression in Jurkat TAg cells**. A. Jurkat cells that express the large T Ag were transiently transfected by electroporation with an IL-2 promoter-luciferase construct. Co-stimulation of these T-cells with 1 μM ionomycin and 10 ng/ml PMA for ~6 hr resulted in 6.4 ± 0.4 – fold increase in IL-2 promoter-driven luciferase reporter expression relative to control non-stimulated cells (*P *= 0.002). B. Similar co-stimulation of Jurkat T Ag cells that were transfected with NFAT3 promoter-luciferase construct reulsted 14.6 ± 1.3 – fold increase in NFAT3 promoter-driven luciferase expression relative to control (*P *= 0.008).

We compared the transcriptional capacity of the full-length IL-2 promoter with that of a synthetic promoter composed of 3 NFAT elements in transient transfection assays using plasmids harboring either one of the two promoters upstream of a luciferase reporter to determine which would allow for more stringent T-cell activation-dependent reporter expression. Jurkat T-cells expressing the large T Ag were transfected with IL-2 promoter-luciferase plasmid (Figure 1A) or NFAT3 promoter-luciferase plasmid (Figure 1B). Co-stimulation of transfected cells with 1 μM ionomycin and 10 ng/ml PMA for ~6 hr resulted in 6.4 ± 0.4 – fold increase in IL-2 promoter-driven luciferase reporter expression (*P *= 0.002) and 14.6 ± 1.3 – fold increase in NFAT3 promoter-driven luciferase expression (*P *= 0.008) which were both significant relative to transfected untreated cells or cells stimulated with either drug alone (n = 3). Although T-cell activation resulted in an average 2.3 ± 0.2 – fold stronger induction capacity of luciferase reporter expression from the NFAT3 promoter compared to the IL-2 promoter (*P *< 0.001), the basal transcriptional activity of the NFAT3 promoter in the absence of T-cell activation was 4.6 ± 0.2 – fold higher than that of the IL-2 promoter (*P *< 0.001). These results indicated that the IL-2 promoter serves as more stringent T-cell activation-dependent promoter than the synthetic NFAT3 promoter.

### Synthesis of the SINIL-2pr retrovector

**Figure 2:**
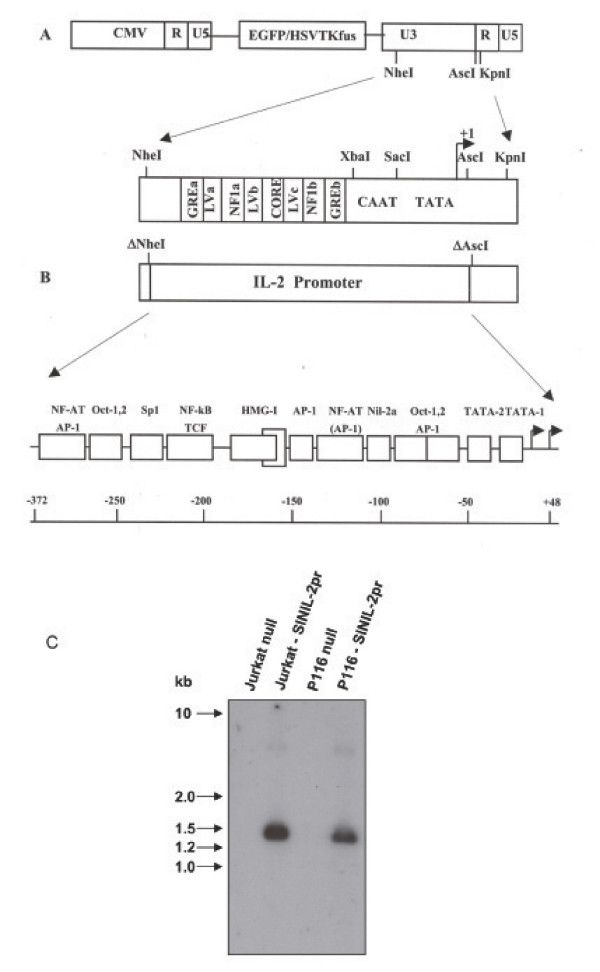
**Design of the SINIL-2pr retrovector**. A. pGFP/TKfus plasmid bears a full-length 3'LTR that incorporates all the promoter and enhancer machinery intrinsic to the wild-type MoMLV retrovirus. The CMV promoter element which substitutes the U3 region in the 5' LTR drives the expression of the retroviral genome in transfected packaging cells for the production of replication-defective retrovirus. B. SINIL-2 pr retrovector is designed by creating an NheI-AscI deletion to the 3'LTR of pGFP/TKfus and by replacing the U3 with the full-length human IL-2 promoter which results in a self-inactivating retrovector whereby expression of the EGFP and HSVTK fusion protein is dependent on the IL-2 promoter in cells transduced with SINIL-2pr retroparticles. C. Southern Blot analysis of the integrated proviral DNA in Jurkat and p116 cells transduced with SINIL-2pr retrovector at similar MOI. Genomic DNA was extracted from transduced and control null cells, digested with KpnI and probed with [P^32^]-labeled probe complementary to the GFP reporter cDNA. The detected DNA bands are consistent with the predicted sized fragment indicating no rearrangement in the integrated proviral DNA.

To develop a retroviral vector for activation-induced stable transgene expression in T-cells, we generated a self-inactivating retroviral vector (SIN) by creating extensive deletions to the U3 region of the Moloney Murine Leukemia Virus 3' long terminal repeat (3'LTR) in pGFP/TKfus (Figure 2A). Retroviral promoter and enhancer machinery were replaced by the full-length human IL-2 promoter as described in the methods section. The resulting construct SINIL-2pr incorporated the EGFP fused to HSVTK as a reporter/suicide gene (Figure 2B). Subsequently, the SINIL-2pr plasmid was stably transfected into the 293GPG packaging cell line to generate polyclonal as well as single clone retroviral producer populations. Vesicular Stomatitis Virus-G Protein (VSV-G) pseudotyped retroparticles were produced and used to transduce the Jurkat T-cell line and the ZAP-70-deficient Jurkat P116 T-cell line. Since recombinant retroviral vectors can be be susceptible to rearrangements prior to their final integration as a DNA proviral genome, we characterized the integrated conformation in transduced target cells by Southern Blot analysis (Figure 2C). Genomic DNA was extracted from null & from transduced Jurkat cells and P116 cells, digested with KpnI and probed with [P^32^]-labeled DNA sequences complementary to the GFP reporter cDNA. We detected DNA bands consistent with the predicted sized fragment expected from KpnI digest of integrated unrearranged proviral DNA.

### Cyclosporin-A-sensitive activation-induced EGFP expression in Jurkat cells transduced with SINIL-2pr retroparticles

**Figure 3:**
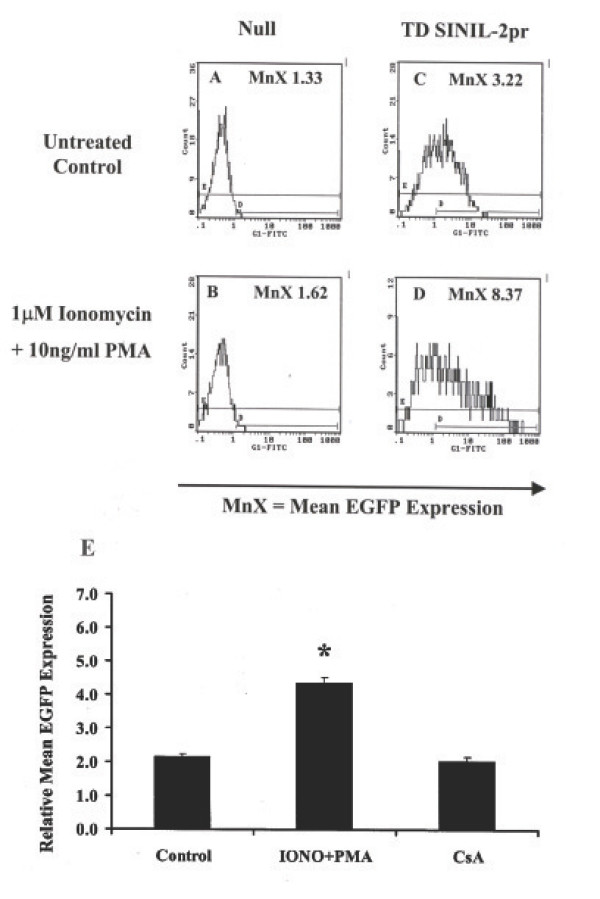
**Activation-induced EGFP expression in Jurkat cells transduced with SINIL-2pr retroparticles**. A-D. Flow cytometry analysis of mean EGFP expression in Jurkat T-cells transduced with SINIL-2pr retroparticles relative to Jurkat null cells. Expression was measured at ~48 hr post-activation with 1 μM ionomycin and 10 ng/ml PMA relative to untreated cells. E. Cyclosporin A-sensitive induction of EGFP expression in Jurkat cells transduced with SINIL-2pr. Co-stimulation of the SINIL-2pr-gene modified Jurkat T-cells with 1 μM ionomycin and 10 ng/ml PMA resulted in a 2.0 ± 0.1 – fold increase in relative mean EGFP expression measured at ~48 hrs (*P *= 0.011). This activation-induced EGFP expression was abrogated when the cells were pretreated for ~30 minutes with 300 nM CsA.

In order to examine whether the SINIL-2pr configuration leads to activation-induced expression in Jurkat T-cells, we compared the level of EGFP reporter expression in Jurkat T-cells transduced with SINIL-2pr retroparticles at a multiplicity of infection (MOI) of 5, (three consecutive transduction rounds) following drug stimulation relative to that of untreated cells (Figure 3). Mean EGFP expression (MnX) was quantified at ~48 hr post -activation using flow cytometry. Figure 3C shows the flow cytometry profile of one representative experiment whereby a mixed population of SINIL-2pr-transduced Jurkat T-cells had detectable EGFP fluorescence (MnX = 3.22) relative to control non gene-modified cells (MnX = 1.33, Figure 3A). Since the SINIL-2pr design is expected to display a "self-inactivation" phenotype with no expression driven from the incorporated IL-2 promoter in the absence of any T-cell activation, we speculated that this low level of EGFP expression resulted from the basal level of activation in Jurkat T-cells since these cells are dividing continuously in culture. Co-stimulation of the mixed SINIL-2pr-transduced population with 1 μM ionomycin and 10 ng/ml PMA for ~6 hr resulted in enhanced EGFP reporter expression (MnX = 8.37, Figure 3D) as compared to stimulated null cells (MnX = 1.62, Figure 3B). Results obtained from three independent experiments (Figure 3E) showed an average 2.0 ± 0.1 – fold increase (mean ± SEM) in relative mean EGFP expression in SINIL-2pr – transduced Jurkat cells following ionomycin and PMA treatment which was significantly higher than that in untreated cells (*P *= 0.011). This activation-induced expression was abrogated when the cells were pretreated for 30 minutes with 300 nM CsA, a potent inhibitor of calcineurin and therefore of the signal transduction cascade that leads to IL-2 promoter transcriptional activation, indicating that the enhanced expression of EGFP was under the regulatory control of the IL-2 promoter.

### Cyclosporin A-sensitive activation-induced EGFP expression in Jurkat P116 cells transduced with SINIL-2pr retroparticles

**Figure 4:**
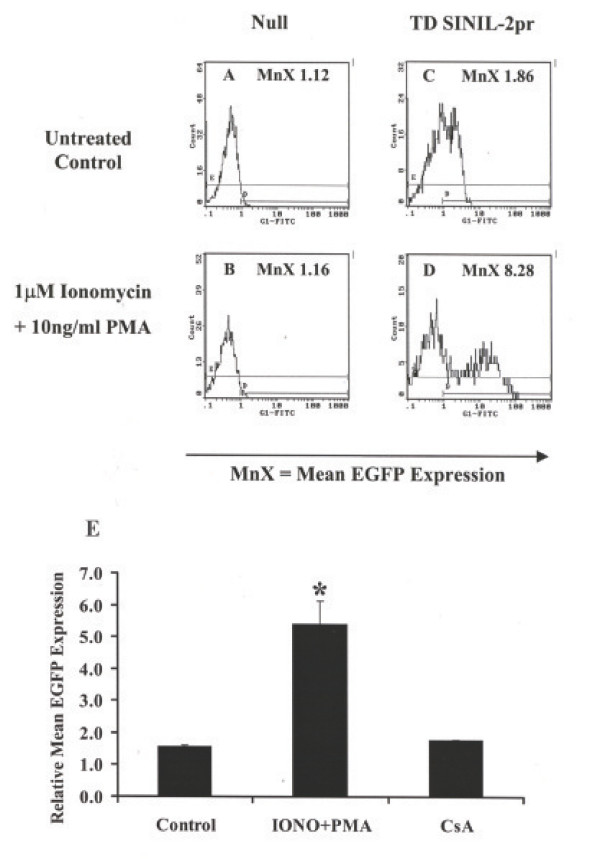
**Activation-induced EGFP expression in Jurkat P116 cells transduced with SINIL-2pr retroparticles**. A-D. Flow cytometry analysis of mean EGFP expression in Jurkat cells deficient for ZAP-70 (Jurkat P116) transduced with SINIL-2pr retroparticles relative to null cells. Expression was measured at ~48 hr post-activation with 1 μM ionomycin and 10 ng/ml PMA relative to untreated counterparts. E. Cyclosporin A-sensitive induction of EGFP expression in Jurkat P116 cells transduced with SINIL-2pr. Co-stimulation of the SINIL-2pr- gene modified Jurkat P116 T-cells with 1 μM ionomycin and 10 ng/ml PMA resulted in a 3.4 ± 0.4 – fold increase in relative mean EGFP expression measured at ~48 hrs (*P *= 0.029). This activation-induced EGFP expression was abrogated when the cells were pretreated for ~30 minutes with 300 nM CsA.

To determine if the low levels of EGFP reporter expression obtained in Jurkat cells transduced with SINIL-2pr prior to drug stimulation were indeed a consequence of basal state of activation in this tumor T-cell line, we tested the SINIL-2pr design in Jurkat P116 T-cells that are deficient in ZAP-70, a kinase implicated in early steps in T-cell activation. Though expandable in culture, Jurkat P116 cells exhibit a longer doubling time compared to that of the parent cell line reflecting a lower basal activation state (data not shown). Figure 4 shows the flow cytometry profile of one representative experiment whereby a mixed polyclonal population of SINIL-2pr-transduced Jurkat P116 T-cells showed level of EGFP fluorescence (MnX = 1.86, Figure 4C) that was similar to background fluorescence of control null cells (MnX = 1.12, Figure 4A). Co-stimulation of the SINIL-2 pr-modified cells with 1 μM ionomycin and 10 ng/ml PMA for ~6 hr resulted in a marked increase in EGFP reporter expression (MnX = 8.28, Figure 4D) as compared to stimulated null cells (MnX = 1.16, Figure 4B). Moreover, following drug stimulation, there was a clear-cut segregation between the EGFP-expressing population and the EGFP-negative population. Results obtained from three independent experiments (Figure 4E) showed an average 3.4 ± 0.4 – fold increase in relative mean EGFP expression in SINIL-2pr – transduced Jurkat P116 cells following ionomycin and PMA treatment which was significantly higher than that in untreated transduced cells (*P *= 0.029). This activation-induced expression was inhibited when the Jurkat P116 cells were pretreated for 30 minutes with 300 nM CsA.

## Discussion

In this study, we first compared the transcriptional potency of two T-cell activation-dependent promoters, the IL-2 promoter and a synthetic promoter composed of 3 repeats of the NFAT element following pharmacological stimulation of transiently transfected T-cells. Plasmid vectors incorporating one of the two promoters were used to transfer luciferase reporter expression into Jurkat T-cells expressing the large T-antigen. Subsequent stimulation of the cells with the drugs ionomycin (a calcium ionophore) and PMA induces polyclonal T-cell activation by causing direct and rapid increase in intracellular [Ca^2+^] [25]. Although the composite NFAT promoter resulted in stronger induction of luciferase reporter expression following T-cell activation, the full-length IL-2 promoter had much lower basal levels of luciferase reporter expression in the absence of any stimulation (Figure 1). These results indicate that unlike the synthetic NFAT promoter, the IL-2 promoter in the plasmid background was strictly controled as there was no considerable constitutive reporter expression in un-stimulated cells. Furthermore, costimulation with ionomycin and PMA resulted in a significant increase in IL-2 promoter-driven luciferase expression compared to unstimulated cells.

It was shown that reporter gene expression driven by the full-length IL-2 promoter in transfected human peripheral blood T lymphocytes and the Jurkat T- cell line paralleled that of the endogenous IL-2 gene following T-cell activation [26]. Their results also indicated that the NFAT sites were less implicated in transcriptional regulation in normal T cells than they were in the tumor T-cell line. A later study showed that activation of NFAT element is insufficient for activation of the IL-2 promoter, and that the NFAT and IL-2 promoters differ in their requirements for co-stimulation [27]. Furthermore, Hooijberg E *et al*., used a self-inactivating retroviral vector in which GFP reporter expression was controlled by multiple (3 or 6) repeats of NFAT elements followed by a minimal IL-2 promoter to visualize and isolate antigen-stimulated Jurkat cells, primary T-cell blasts and antigen-specific T-cell clones [28]. Although the design resulted in activation-induced GFP expression, the authors reported that a percentage of the gene-modified T-cells expressed GFP constitutively independently of activation. In addition, not all T-cells in an antigen-specific clonal population had upregulated GFP expression following activation. The sum of these studies and our results suggest that the full-length IL-2 promoter may serve as a more specific and stringent activation-dependent promoter in T-cells.

Subsequently, we examined the potential of the full-length IL-2 promoter within the context of a retroviral vector to drive stable and activation-induced reporter expression in transduced Jurkat T-cells. The cDNA for human IL-2 promoter was incorporated into a self-inactivating retroviral vector replacing the enhancer and promoter machinery in the 3'LTR of MoMLV. The resulting SINIL-2pr construct incorporated EGFP fused to HSVTK as a reporter/suicide gene (Figure 2). VSV-G pseudotyped retroparticles were generated and were used to transduce the Jurkat T-cell line. Flow cytometry analysis showed that expression of the EGFP reporter was markedly enhanced post co-stimulation of the gene-modified Jurkat T-cells with ionomycin and PMA (Figure 3). Furthermore, activation-induced EGFP expression from the SINIL-2 design was specific to induction of the IL-2 promoter as it was sensitive to pre-treatment of the cells with cyclosporin A (Figure 3E), an immunosuppressant drug that blocks IL-2 promoter transcriptional activation by interfering with the enzymatic activity of the phosphatase calcineurin [29].

However, although there was no significant reporter expression from the IL-2 promoter in the plasmid backbone, the SINIL-2 pr design did result in measurable EGFP expression in unstimulated transduced Jurkat cells. We speculated that this basal level of EGFP expression reflects a basal status of activation in the Jurkat tumor T-cell line since the cells are constantly dividing in culture that is not the case for unactivated primary T-cells that are quiescent in the absence of stimulation. To investigate this we transduced the Jurkat P116 T-cells. Due to their defectiveness in ZAP-70, a kinase involved in early steps of TCR signaling, the cells divide at a much slower rate compared to the parent cell line reflecting a lower basal status of activation. However, since T-cell activation by co-stimulation with ionomycin and PMA bypasses these early steps, Jurkat P116 cells can be activated with these drugs. Interestingly, the Jurkat P116 cells transduced with SINIL-2 pr had lower basal levels of EGFP expression compared to gene-modified Jurkat cells (Figure 4). Furthermore, as speculated, the mutant cells resulted in a stronger induction of reporter expression following drug stimulation whereby the segregation between the GFP +ve and the GFP -ve cells was more evident.

Zhang P-X and Fuleihan RL used a recombinant adeno-associated virus (AAV) vector incorporating the IL-2 promoter to drive activation-dependent luciferase reporter expression in the Jurkat T-cell line [30]. However, there was low efficiency of gene transfer with very few clonal AAV integration events. Despite *in vitro *selection in G418, some of the transferred material was lost or silenced over time. Furthermore, to induce luciferase reporter expression following T-cell activation, the cells had to be subjected to additional stimulation such as heat shock and irradiation due to the nature of the vector used. Hence, taking all this into consideration, the AAV system would be greatly limited if applied to primary T-cells.

Since cytotoxic CD8+ T-cells do not significantly induce IL-2 promoter as compared to CD4+ T-cells, we propose that the SINIL-2pr system would be especially useful in applications such as negative selection of alloreactive CD4+ T-cells when the disparity between donor and recipient is at the MHC Class II locus and for preferential enrichment of antigen-specific CD4+ T-cells which have been shown to confer an advantage in several immunotherapy applications. In one study, unselected donor T-lymphocytes with a predominance of CD4+ population resulted in a stronger expansion of virus-specific T-cells [31]. Similarly, another study showed that there was a strikingly higher proportion of CD4+ EBV-specific T-cells transduced after early sensitization with EBV-presenting B-cells [19]. In adoptive immunotherapy of cancer, CD4+ T-cells were also shown to play an essential role by providing early stimuli for proliferation of tumor-reactive T-cells [32]. In addition, co-expression of the EGFP reporter and the HSVTK suicide gene as a fusion protein in the SINIL-2pr design allows not only for selection of the engineered T-cells but also conditional destruction following GCV treatment. The utility of the EGFP-HSVTK fusion as a "bifunctional" reporter/suicide transgene and its potential use in primary human T-cells was already demonstrated in previously published work by our group [33].

## Conclusion

We conclude that the SINIL-2 pr design can serve as platform for stable, specific, and activation-induced expression in T-cell lines. Further experiments need to be done to investigate the function of this system in primary T-cells and to exploit its use in several T-cell immunotherapy applications such as negative selection of alloreactive T-cells and their elimination from the donor T-cell population prior to infusion or for *ex vivo *sorting of antigen-specific T-cells and their subsequent *in vivo *tracking.

## Methods

### Cell lines and plasmids

The IL-2 promoter-luciferase plasmid (IL-2Luc) and the NFAT3 promoter-luciferase plasmid (NFATLuc) were previously described [34]. The Jurkat human acute lymphoblastic leukemia T-cell line was obtained from the American Type Culture Collection (ATCC, Rockville, MD). Jurkat cells expressing the SV40 large T antigen (Jurkat TAg) were previously described [34]. ZAP-70-deficient Jurkat P116 cells were generously provided by Dr. R. T. Abraham (Department of Immunology, Mayo Clinic, Minnesota). All T-cell lines were maintained in RPMI media (Gibco-BRL, Gaithesburg, MD) supplemented with 10% heat-inactivated FBS (Gibco-BRL) and 1% penicillin-streptomycin. pJ6Ω Bleo plasmid and 293GPG retroviral packaging cell line were generous gifts from Dr. Richard. C. Mulligan (Children's Hospital, Boston, MA). 293GPG cells were maintained in media composed of DMEM (Gibco-BRL), 10% heat-inactivated FBS supplemented with 0.3 mg/ml G418 (Mediatech, Herndon, VA), 2 μg/ml puromycin (Sigma, Oakville, ONT), and 1 μg/ml tetracycline (Fisher Scientific, Nepean, ONT).

### Transfection of Jurkat TAg cells and drug stimulation

20 × 10^6 ^Jurkat T-cells expressing the large T antigen were co-transfected with a total of ~10 μg DNA of either the IL-2 promoter-luciferase or the NFAT3 promoter-luciferase plasmid and PEF-GFP (empty vector) at a ratio of 10:1. A control sample was transfected with ~10 μg PEF-GFP DNA only to normalize for the efficiency of transfection. Transient transfections were performed using standard electroporation at 960 μF and 240 V (Bio-Rad, Hercules, CA). The samples were then cultured in 75-cm^2 ^tissue culture flasks in complete RPMI media at 37°C and 5%CO2 for two days. Afterwards, samples were analyzed by flow cytometry (FACStar sorter, Becton Dickinson, Mountain View, CA) to determine transfection efficiency based on EGFP fluorescence. Each sample was then divided into 4 subsets that were either stimulated with 1 μM ionomycin (Sigma, St. Louis, MO) or/and 10 ng/ml PMA (Sigma) or left untreated for ~6 hr.

### Luciferase assays

Transfected Jurkat TAg cells were harvested ~6 hr post stimulation and washed twice with phosphate-buffered saline (PBS). Afterwards, cells in each sample were lysed using 250 μl lysis buffer (Luciferase Reporter Gene Assay, Boehringer Mannheim). Then, a 20 μl aliquot of each lysate supernatant was mixed with 50 μl luciferase reagent buffer containing the luciferin substrate (Luciferase Reporter Gene Assay, Boehringer Mannheim). Within 30 sec of starting the reaction per sample, luciferase activity was measured as light units based on light emission at 562 nm using the Lumat LB-9507 luminometer (Perkin Elmer Instruments, Rodgau-Juegesheim, Germany) according to manufacturer's specifications. All measurements were done in triplicates.

### SINIL-2pr retrovector design and synthesis

We used a derivative of pGFP/TKfus [33] to generate the SINIL-2pr design. pGFP/TKfus contains the cDNA for the enhanced green fluorescent protein (EGFP) reporter and the herpes simplex virus thymidine kinase (HSVTK) suicide gene as a fusion and incorporates the CMV promoter in the 5'LTR which drives expression in transfected viral packaging cells. We derived a self-inactivating (SIN) vector from this plasmid by creating a 341-bp NheI-AscI/Klenow deletion in the 3'LTR to remove all the retroviral promoter and enhancer machinery. An insert encoding for the human IL-2 promoter (-372 to +48) was generated by PCR (PTC-100™ Programmable Thermal Controller, MJ Research Inc.) using CTA GCT AGC (introducing NheI site) GAA AGG AGG AAA AAC TGT TTC AT sense primer and C GAG CTC (introducing Ecl136II site) TAG GGA AC T CTT GAA CAA GAG antisense primer (Sheldon Biotechnology Centre, McGill University, Montreal). The amplified insert was then digested with the corresponding enzymes, purified, and ligated into the SIN-derivative in order to generate the SINIL-2pr plasmid (Figure 2). Nucleotide sequences of the mutated 3'LTR and the inserted IL-2 promoter were confirmed by DNA sequencing (GenAlyTic Inc., University of Guelph, Ontario).

### Generation of the SINIL-2pr retroviral producers and production of VSV-G pseudotyped retroviral particles

The SINIL-2pr retroviral producers were generated by stable transfection of the 293GPG packaging cell line as previously described [35]. In brief, stable producer cells were generated by co-transfection of 5 μg FspI-linearized SINIL-2pr retrovector and pJ6Ω Bleo plasmid at a 10:1 ratio. Transfected packaging cells were subsequently selected in 293GPG media supplemented with 100 μg/ml Zeocin (Invitrogen, San Diego, CA) for 3–4 weeks. Resulting stable polyclonal as well as isolated single clone producer populations were utilized to generate VSV-G pseudotyped retroviral particles. All viral supernatants were filtered through 0.45-μm pore size syringe-mounted filters (Gelman Sciences, Ann Arbor, MI) and stored at -20°C. Subsequent 100 × concentration of the retroviral preparations was performed using ultracentrifugation according to a previously described procedure [35] after which samples were stored at -80°C.

### SINIL-2pr viral titer determination and RCR assay

Jurkat T-cells were suspension-cultured in 12-well plates at ~2 × 10^5 ^cells per well per 1 ml of the concentrated retroviral sample serially diluted in complete RPMI media supplemented with 6 μg/ml Polybrene (Sigma). Cells transduced with the various viral dilutions were then incubated overnight at 37°C and 5 % CO_2_. Afterwards, the samples were spun at 1000 g for 5 min to pellet the cells and the virus-containing supernatent was discarded. The cells for each sample were re-suspended in 2 ml fresh complete RPMI media and expanded in culture. FACS analysis was performed on these samples on 5^th ^day post transduction to ascertain retrovector expression and gene transfer efficiency as measured by EGFP fluorescence. Based on the number of target cells per well per dilution sample at the time of viral addition and assuming that each cell takes 1 retroparticle when the gene transfer is not saturated (less than 40%), the viral titer was estimated as ~1 × 10^7 ^infectious particles per ml. Viral preparations were devoid of replication competent retrovirus (RCR) as determined by standard EGFP marker rescue assay performed on null untransduced Jurkat cells exposed to conditioned supernatant collected from transduced Jurkat cells.

### Transduction of Jurkat and P116 T-cell lines, Southern Blot, and drug stimulation

Each of Jurkat and P116 T-cell lines was suspension-cultured at ~2 × 10^5 ^cells per well per 1 ml of complete RPMI media. The cells were transduced with SINIL-2pr concentrated retrovirus at an MOI of 5 in whole media supplemented with 6 μg/ml Polybrene. This procedure was repeated daily for ~6 hr exposure for three consecutive days after which stably transduced cells were expanded. No clonal selection was performed, and mixed populations of transduced cells were used for all subsequent experiments. Southern blot analysis was performed on 15 μg of overnight KpnI digested genomic DNA extracted from stably transduced cells as well as untransduced control cells. Blots were hybridized with P^32 ^labeled cDNA probes, washed and exposed on photographic film. T-cell stimulation was done using 1 μM ionomycin and/or 10 ng/ml PMA in whole media for ~6 hr and in some experiments following 30 min exposure to 300 nM cyclosporin A (CsA) (Novartis Pharma Canada Inc.). FACS analysis was performed to ascertain retrovector expression as measured by mean EGFP fluorescence of positively gated cells.

### Statistics

Student T test was applied using Microsoft Excel software.

## Competing interests

The author(s) declare that they have no competing interests.

## Authors' contributions

DJ carried out the cloning and generation of the various constructs, retroviral production and titer assays, transduction and subsequent analysis of target cells, activation assays, luciferase assays, data acquisition and analysis, and drafted the manuscript. LL did the Southern Blot, and revised the manuscript. CC contributed to the conception of the designs in the study, acquisition of funding, and revised the manuscript. JG had substantial contribution to the conception of the study, the experimental designs, general supervision of the research, acquisition of funding, and critically revised the manuscript. All authors approved of the final manuscript version.
